# Study of ZrO_2_ Gate Dielectric with Thin SiO_2_ Interfacial Layer in 4H-SiC Trench MOS Capacitors

**DOI:** 10.3390/ma18081741

**Published:** 2025-04-10

**Authors:** Qimin Huang, Yunduo Guo, Anfeng Wang, Zhaopeng Bai, Lin Gu, Zhenyu Wang, Chengxi Ding, Yi Shen, Hongping Ma, Qingchun Zhang

**Affiliations:** 1 Institute of Wide Bandgap Semiconductors and Future Lighting, Academy for Engineering & Technology, Fudan University, Shanghai 200433, China; 2 Shanghai Research Center for Silicon Carbide Power Devices Engineering & Technology, Fudan University, Shanghai 200433, China; 3 Institute of Wide Bandgap Semiconductor Materials and Devices, Research Institute of Fudan University in Ningbo, Ningbo 315327, China

**Keywords:** SiO_2_/ZrO_2_, ZrO_2_, trench structure, ALD, gate dielectric, MOS capacitor

## Abstract

The transition of SiC MOSFET structure from planar to trench-based architectures requires the optimization of gate dielectric layers to improve device performance. This study utilizes a range of characterization techniques to explore the interfacial properties of ZrO_2_ and SiO_2_/ZrO_2_ gate dielectric films, grown via atomic layer deposition (ALD) in SiC epitaxial trench structures to assess their performance and suitability for device applications. Scanning electron microscopy (SEM) and atomic force microscopy (AFM) measurements showed the deposition of smooth film morphologies with roughness below 1 nm for both ZrO_2_ and SiO_2_/ZrO_2_ gate dielectrics, while SE measurements revealed comparable physical thicknesses of 40.73 nm for ZrO_2_ and 41.55 nm for SiO_2_/ZrO_2_. X-ray photoelectron spectroscopy (XPS) shows that in SiO_2_/ZrO_2_ thin films, the binding energies of Zr 3d_5/2_ and Zr 3d_3/2_ peaks shift upward compared to pure ZrO_2_. Electrical characterization showed an enhancement of *E*_BR_ (3.76 to 5.78 MV·cm^−1^) and a decrease of *I*_ON_EBR_ (1.94 to 2.09 × 10^−3^ A·cm^−2^) for the SiO_2_/ZrO_2_ stacks. Conduction mechanism analysis identified suppressed Schottky emission in the stacked film. This indicates that the incorporation of a thin SiO_2_ layer effectively mitigates the small bandgap offset, enhances the breakdown electric field, reduces leakage current, and improves device performance.

## 1. Introduction

The current SiC MOSFET market is experiencing a new phase of growth, fueled by the rapid expansion of the renewable energy sector [1]. In this context, high-performance SiC MOSFETs are progressively replacing traditional silicon-based power devices [2]. Among materials, SiC has attracted increasing interest in power electronics and renewable energy conversion systems owing to its low on-resistance, high voltage resistance, high-frequency switching capability, and excellent high-temperature reliability [3]. The evolution of SiC MOSFETs from traditional planar to trench-based architectures has led to notable performance improvements, such as higher current density, reduced on-resistance, faster switching speeds, and enhanced blocking voltages [4,5]. However, the introduction of trench structures has also brought technical challenges to the gate dielectric layer [6,7]. Advanced methods such as electron holographic tomography can now be used to study the internal device potential [8]. Despite the high critical breakdown electric field of SiC, the electric field concentrations at the trench corners may subject the oxide layer to excessive stress, severely impacting device reliability and stability [8,9,10]. Therefore, optimizing the design of the gate oxide layer and addressing interface engineering challenges in trench structures have become critical research and development priorities in the field of SiC devices [11,12,13].

Various alternative gate dielectric materials have so far been explored to address these challenges [14]. Wirths et al. demonstrated that integrating high-k dielectrics in place of traditional SiO_2_ in planar SiC MOSFETs effectively mitigates interface trap density, stabilizes threshold voltage behavior, and enhances operational reliability, thereby advancing device performance [15,16,17]. Among high-k materials, Al_2_O_3_, including certain ALON compounds, has been thoroughly investigated [18,19,20,21]. However, zirconia (ZrO_2_) has emerged as a promising candidate due to its high dielectric constant and wide bandgap (5.8 eV) [18]. In this view, Wang et al. investigated the impact of inserting a thin SiO_2_ interlayer between ZrO_2_ and SiC using first-principles calculations, predicting that such a structure could increase band offset, reduce leakage current, and preserve the dielectric advantages of ZrO_2_ [22]. However, the traditional thermal oxidation process of SiC/SiO_2_ interfaces often results in the low density of interface states (*D*_it_), reducing channel mobility [23]. This problem can be solved by optimizing interface engineering to lower interface trap density as a critical focus.

In this study, high-performance gate oxide layers with exceptional uniformity were synthesized using atomic layer deposition (ALD) for the comparative assessment of interfacial characteristics in ZrO_2_-based and SiO_2_/ZrO_2_ trench capacitors. Compared to previous studies, this work provides a more complete experimental assessment of SiO_2_/ZrO_2_ gate dielectric stacks in trench MOS structures. The trench depth, width, and the growth of high-k thin films were studied by scanning electron microscopy (SEM). Comprehensive material validation was performed through multi-technique analysis, including surface morphology examination with atomic force microscopy (AFM), optoelectronic property evaluation by spectroscopic ellipsometry (SE), and chemical composition analysis via X-ray photoelectron spectroscopy (XPS). SE analysis provided critical data on layer thickness and optical bandgap, revealing that the integration of SiO_2_ interfacial layers systematically modulated the energy band structure. Key electrical parameters, such as the dielectric breakdown field intensity (*E*_BR_) and conduction current at the breakdown (*I*_ON_EBR_), were quantified through current–voltage (I-V) profiling. Additionally, capacitance–voltage (C-V) hysteresis analysis allowed for precise determination of D_it_ and effective oxide charge (*N*_eff_), while further investigation of leakage current mechanisms was conducted through I-V curve modeling, providing a detailed understanding of the dominant transport pathways.

Compared to ZrO_2_ alone, the fabricated SiO_2_/ZrO_2_ stacked dielectric exhibited a higher *E*_BR_ and lower *I*_ON_EBR_, underscoring the feasibility and potential of SiO_2_/ZrO_2_ stacked dielectrics as a promising solution for gate oxide applications in trench-structured SiC MOSFETs.

## 2. Experimental Section

**Figure 1:**
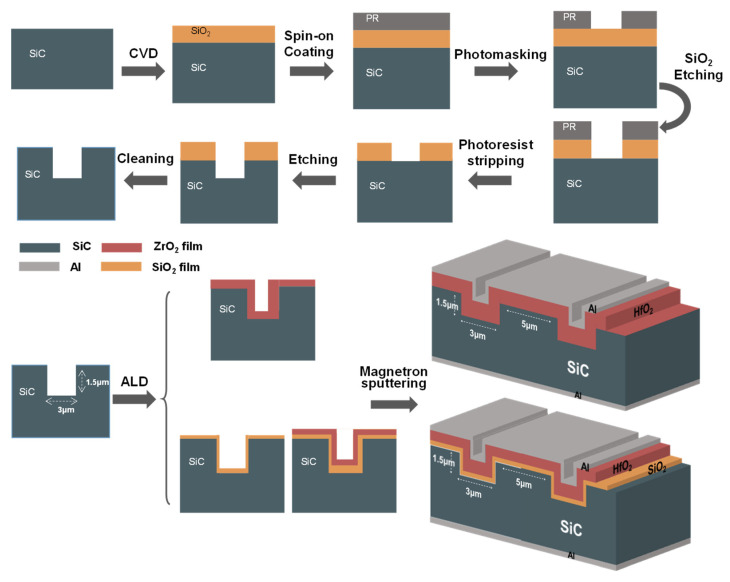
Fabrication process flow and final trench MOS device structure.

The sample preparation process of the films and a scheme of the final product are depicted in Figure 1. The experiment commenced with a 4H-SiC epitaxial wafer (n-doped, (0001) orientation with 4° off-axis) as the substrate. A 2.5 µm-thick SiO_2_ masking layer was initially grown via chemical vapor deposition (CVD, SINO-Plasma 8000, amteglobal, Wuxi, China). Next, photolithography (ABM/6/350/NUV/DCCD/M, ABM Inc., San Jose, CA, USA) was employed to pattern the mask, defining trench arrays with 3 µm width. Following SiO_2_ layer patterning through dry etching, the residual photoresist was stripped via solvent treatment, enabling subsequent reactive ion etching of the SiC substrate to define trench architectures. The finalized structures exhibited precise dimensional control with trench widths of 3 µm, vertical depths of 1.5 µm, and inter-trench spacing of 5 µm. To ensure mask integrity, buffered oxide etch (BOE) was selectively applied to remove residual oxides from unpatterned regions.

After the formation of clean trench structures, the gate dielectric layer was deposited using an atomic layer deposition (ALD, Exploiter E200SP, SuperALD, Shenzhen, China) system at 270 °C. For the single-layer ZrO_2_ film, 400 deposition cycles were sufficient. For the SiO_2_/ZrO_2_ stacked dielectrics, sequential deposition of 10 nm SiO_2_ (precursor: plasma) and 30 nm ZrO_2_ (precursor: CpZr(NMe_2_)_3_) resulted in a composite dielectric layer with a total thickness of approximately 40 nm after 400 cycles, demonstrating excellent thickness uniformity. Finally, 200 nm aluminum electrodes were deposited on both device surfaces via magnetron sputtering, with standardized 200 × 200 μm^2^ test contacts formed. A schematic representation of the obtained ZrO_2_ thin film and SiO_2_/ZrO_2_ stacked film samples is also shown in Figure 1.

Following etching and ALD, the cleaved samples were fixed onto the sample stage using conductive adhesive. To improve conductivity, a thin layer of gold was applied to the sample surface using sputtering for 30 s. The cross sections were analyzed using a scanning electron microscope (SEM, Sigma 300, ZEISS, Oberkochen, Germany) to evaluate the trench etching quality and the uniformity of the film deposition within the trench.

Additional analysis of the ALD-deposited films was performed by determining the film thickness through spectroscopic ellipsometry (SE, Woollam RC2, J.A. Woollam, Lincoln, NE, USA). The SiC substrate was characterized using B-spline curves and the Cauchy dispersion model to determine the film thickness and optical parameters, enabling detailed characterization of the gate dielectric layer. The surface morphology was analyzed using atomic force microscopy (AFM, BRUKER BRK0003, Bruker Corporation, Karlsruhe, Germany) over a 5 µm × 5 µm area, with raw data processed by NanoScope analysis (3.0) (Billerica, MA, USA) software to determine surface roughness. X-ray photoelectron spectroscopy (XPS, ESCALAB 250Xi, Thermo Scientific, Waltham, MA, USA) examined the chemical states of the films, with all XPS spectra calibrated to the C 1s peak at a binding energy of 284.8 eV.

The electrical characterization of thin-film devices was systematically performed utilizing a TS2000-HP probe system (MPI Corporation, Hsinchu, Taiwan) coupled with a Keithley 4200A-S parametric analyzer (Tektronix Inc., Beaverton, OR, USA). Current–voltage characteristics were recorded through progressive elevation of the gate bias until dielectric failure occurred, enabling determination of both *E*_BR_ and *I*_ON_EBR_ in metal–oxide–semiconductor structures. Capacitance–voltage profiling was implemented through gate voltage scanning under alternating current excitation (30 mV amplitude) across multiple frequency settings. Subsequent analysis of capacitance–voltage relationships permitted quantitative evaluation of *D*_it_ and *N*_eff_ in the fabricated capacitors. All measurements were performed at room temperature.

## 3. Results

**Figure 2:**
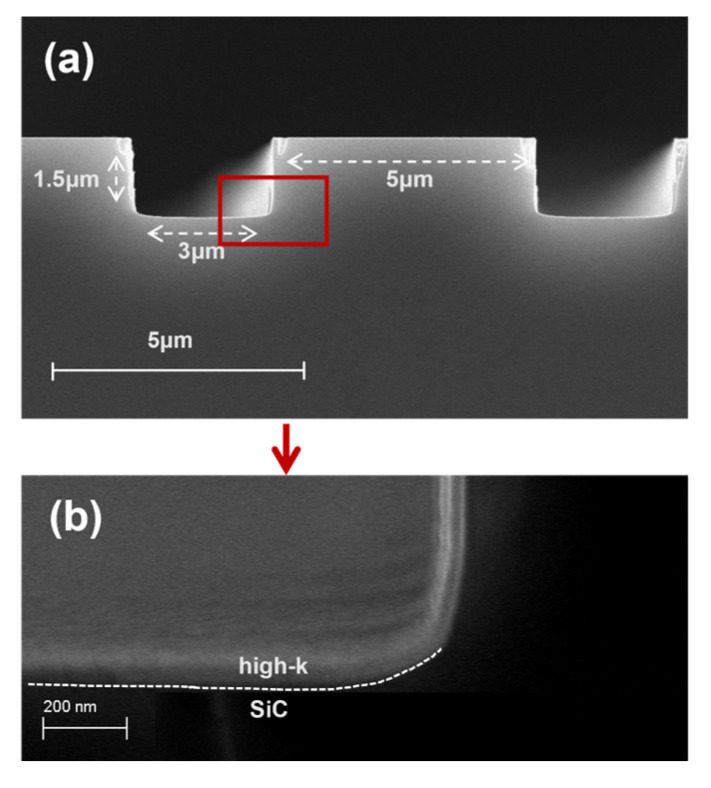
SEM images of (**a**) the trench structure and (**b**) the magnified view of the corner region marked by the red box in (**a**) after the ALD process.

In this study, ZrO_2_ and SiO_2_/ZrO_2_ gate dielectric films were grown by atomic layer deposition, and their characteristics were studied by various analytical techniques. The trench structures observed by SEM in Figure 2a revealed the deposition films with trench widths of 3 µm, vertical depths of 1.5 µm, and a trench pitch of 5 µm. The SEM image in Figure 2b shows a gate oxide layer that was grown using ALD. The high-k layer demonstrated excellent uniformity and consistency across the entire trench surface, even at the trench corners. Thus, the ALD process can be used to deposit films with precisely controlled film thickness and uniformity, outperforming the traditional dry oxidation method.

**Figure 3:**
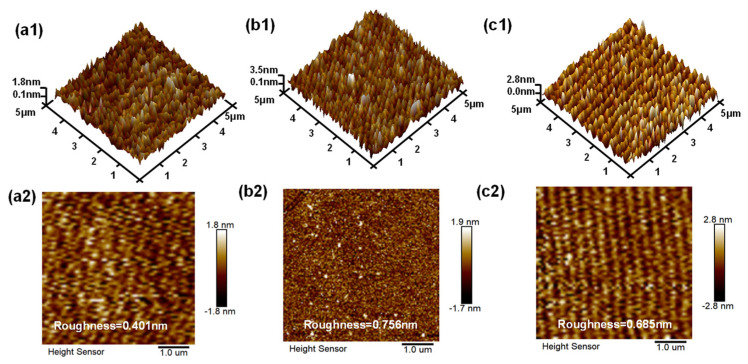
3D and 2D AFM images within a 5 × 5 µm^2^ area are shown for: (**a1**,**a2**) ZrO_2_, (**b1**,**b2**) SiO_2_/ZrO_2_, (**c1**,**c2**) interlayer SiO_2_.

The surface morphologies and roughnesses of ZrO_2_ thin films, SiO_2_/ZrO_2_ stacked films, and the interfacial SiO_2_ layer analyzed by AFM are compared in Figure 3. Both types of films exhibited relatively smooth surfaces, with a roughness value of 0.401 nm for ZrO_2_ thin films and 0.756 nm for SiO_2_/ZrO_2_ stacked films. Further analysis of the surface profiles revealed roughness within a sub-1 nm range, indicating the deposition of films with a smooth surface finish. By comparison, the surface roughness of the SiO_2_/ZrO_2_ stacked films was slightly higher than that of single-layer ZrO_2_ films. Such an increase in roughness can be attributed to the physical and chemical differences between SiO_2_ and ZrO_2_, which might induce interfacial stress or defects during the deposition process. Additionally, the multilayer nature of the stacked structure may have contributed to cumulative roughness since each additional layer introduced a minor increment to the overall surface roughness

**Figure 4:**
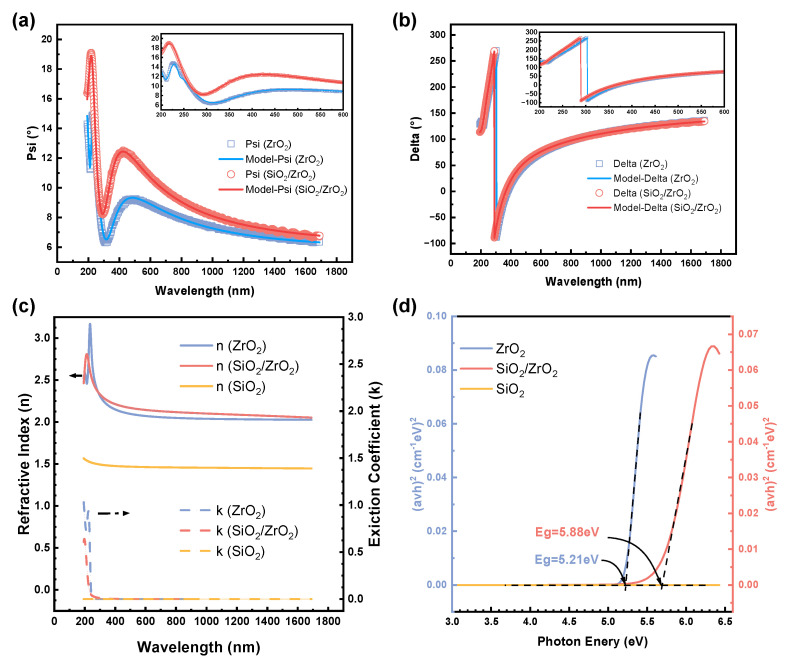
The SE analysis of ZrO_2_ and SiO_2_/ZrO_2_ film heterostructures: (**a**) Ψ and (**b**) Δ components of complex reflectance. (**c**) Refractive index (*n*) and extinction coefficient (*k*) profiles. (**d**) Tauc plot ((*αhν*)^2^ vs. photon energy) for bandgap determination.

Spectroscopic ellipsometry (SE) was employed to systematically analyze multiple regions of the deposited thin films to ensure measurement reliability and determine their thicknesses. The ZrO_2_ single-layer film exhibited an average thickness of 40.73 nm, while the SiO_2_ and ZrO_2_ layers in the SiO_2_/ZrO_2_ bilayer structure measured 10.73 nm and 30.82 nm, respectively. Figure 4a,b present the experimental Ψ and Δ parameters, while the corresponding refractive index (*n*) and extinction coefficient (*k*) are shown in Figure 4c.

The optical bandgap can be determined using the Tauc method, which is based on the relationship (*αhν*)^2^ ∝ (*hν* − *E*_g_), where α is the absorption coefficient, ν is photon frequency, and *E*_g_ is the optical bandgap [24]. This method relies on the fact that, for semiconductors, most electrons in the valence and conduction bands are located near the bandgap. When the energy of incident photons approaches the bandgap, electrons can absorb the photon energy. In this method, the absorption coefficient (α) is calculated using the relationship α = 4πk/λ, where λ is the wavelength of the incident light. The results are displayed in Figure 4. The optical bandgap is extracted by plotting (*αhν*)^2^ versus the photon energy (*hν*) and then linearly extrapolating the results to zero [25].

Using this approach, the optical bandgaps of the ZrO_2_ and SiO_2_/ZrO_2_ composite films were determined to be 5.21 eV and 5.88 eV, respectively [26]. Despite the relatively thin SiO_2_ layer, its inherent high bandgap (approximately 9 eV) contributed to bandgap tuning in the composite film, thereby enhancing the device’s breakdown voltage and reducing leakage current. Therefore, the presence of the SiO_2_ buffer layer in the stack structure can significantly enhance the overall device performance by adjusting the bandgap [27,28].

**Figure 5:**
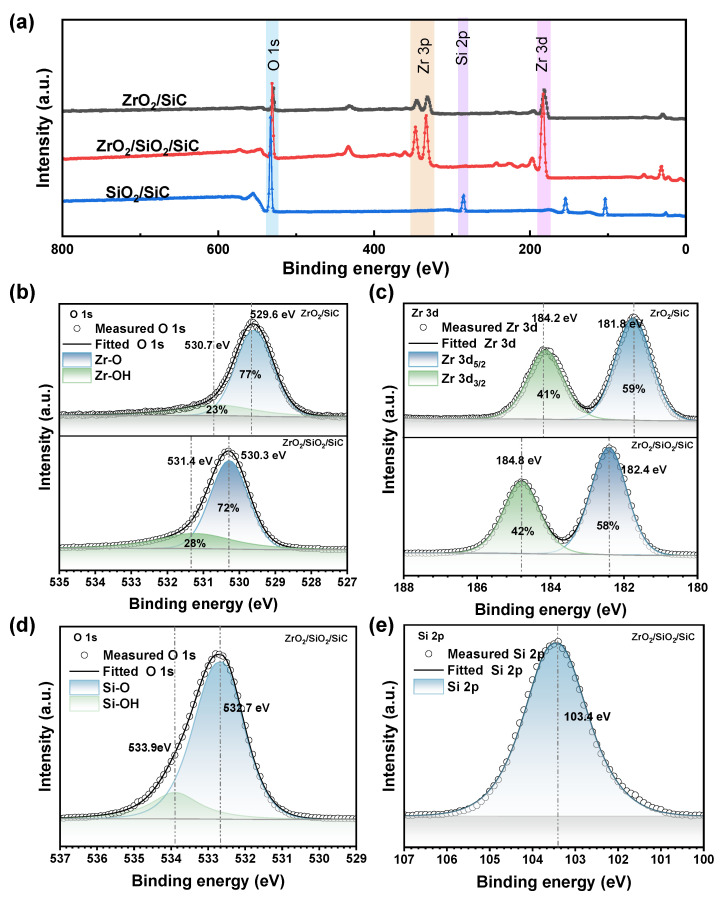
(**a**) XPS spectra of ZrO_2_ and SiO_2_/ZrO_2_, (**b**) O 1s core level and (**c**) Zr 3d core level spectra of ZrO_2_ and SiO_2_/ZrO_2_, (**d**) O 1s core level and (**e**) Si 2p core level spectra of the intermediate SiO_2_ layer.

XPS analysis was employed to probe the elemental composition and chemical bonding states of the dielectric surfaces. Figure 5a displays the full-range XPS spectra for both ZrO_2_ and SiO_2_/ZrO_2_ films. The O 1s core-level XPS spectra and peak deconvolutions for both thin films are presented in Figure 5b. For the ZrO_2_ thin film, the O 1s peak can be deconvoluted into two components at 529.6 eV and 530.7 eV. By comparison, the O 1s peak of SiO_2_/ZrO_2_ thin film can be deconvoluted into two components at 530.3 eV and 531.4 eV.

As shown in Figure 5c, he Zr 3d core-level XPS spectra exhibit well-resolved spin–orbit doublets (Zr 3d_5/2_ and Zr 3d_3/2_) for both ZrO_2_ and SiO_2_/ZrO_2_ thin films, with a characteristic splitting energy of 2.4 eV. For the pristine ZrO_2_ film, the binding energies of Zr 3d_5/2_ and Zr 3d_3/2_ were determined to be 181.8 eV and 184.2 eV, respectively [29]. Upon SiO_2_ interlayer incorporation, these values showed a systematic positive shift to 182.4 eV and 184.8 eV. The experimentally observed area ratio between Zr 3d_5/2_ and Zr 3d_3/2_ peaks was calculated to be 59:41 for ZrO_2_ and 58:42 for SiO_2_/ZrO_2_, closely approximating the theoretical 3:2 ratio dictated by spin–orbit coupling effects [30]. This ratio is consistent with the expected energy level splitting determined by the spin–orbit coupling effect. Spin–orbit coupling, which is the interaction between the spin angular momentum and the orbital angular momentum of an electron, causes energy level splitting. In this case, the spin–orbit coupling of the Zr 3d orbitals results in a consistent area ratio of the Zr 3d_5/2_ and Zr 3d_3/2_ peaks, reflecting the similar electronic states of Zr atoms in both samples.

The O 1s core-level and Si 2p XPS spectra of the SiO_2_ interlayer, along with their peak deconvolutions, are presented in Figure 5d,e. The O 1s peak can be deconvoluted into two components at 532.7 eV and 533.9 eV [31]. The stronger covalent bonding in SiO_2_ may have resulted in a higher electron cloud density around oxygen atoms, leading to higher binding energy of the O 1s peak when compared to that in ZrO_2_.

Overall, the XPS results demonstrate that in the SiO_2_/ZrO_2_ thin film, the binding energies of both the Zr 3d_5/2_ and Zr 3d_3/2_ peaks are shifted upward compared to those in pure ZrO_2_. This indicates that charge redistribution or polarization at the interface has altered the electronic environment of the Zr atoms, leading to an increase in their binding energies.

**Figure 6:**
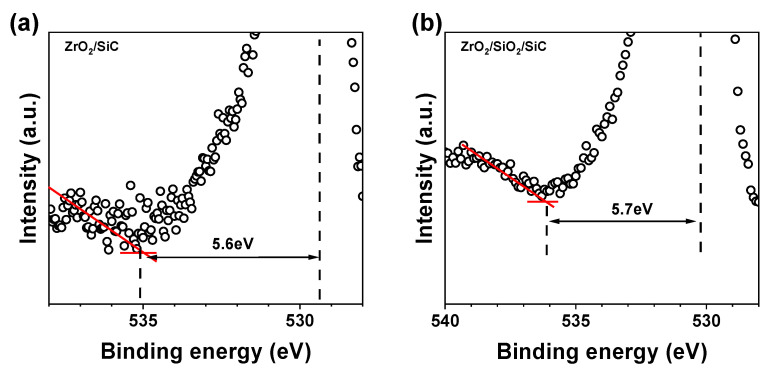
O 1s energy loss spectrum for fitting the band gap of (**a**) ZrO_2_ and (**b**) SiO_2_/ZrO_2_.

As illustrated in Figure 6a,b, the O 1s energy loss spectra (ELS) for both ZrO_2_ and SiO_2_/ZrO_2_ were analyzed by examining the energy differences between the O 1s peak positions. This approach enables the determination of the material’s band gap, as the onset of energy loss corresponds to the excitation of electrons from the valence band to the conduction band. The energy separation between the O 1s peak and the threshold energy loss provides an estimate of the band gap. Using this method, the band gaps were found to be 5.6 eV for ZrO_2_ and 5.7 eV for SiO_2_/ZrO_2_ [32]. The discrepancy between *E*_g_ values derived from ELS and Tauc methods can be attributed to their distinct probing mechanisms. ELS emphasizes localized electronic transitions near the surface, while Tauc analysis reflects bulk optical absorption. These results align with the optical bandgap values obtained from previous fitting analysis.

**Figure 7:**
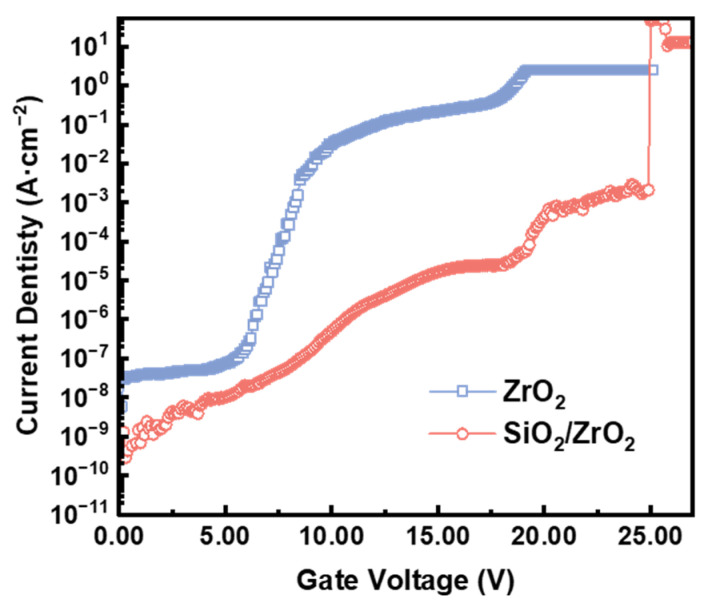
J-V curves of MOS capacitors.

The comparative current density–voltage (J-V) characteristics of ZrO_2_-based MOS capacitors and their SiO_2_/ZrO_2_ stacked counterparts are illustrated in Figure 7. Current density values were normalized to the square electrode area using the relationship $J=\frac{I}{A}$, where A donates the electrode area and I represents the measured current [33]. The J-V curves of both devices exhibited plateaus at lower voltages, followed by a gradual increase as the electric field intensified. However, no distinct breakdown phenomenon was observed for the single-layer ZrO_2_ capacitor, even at higher electric fields. Interestingly, the conduction current stabilized at sustained levels, displaying typical features of a metastable “soft breakdown” state [34]. The analysis revealed that the single-layer ZrO_2_ devices failed at a dielectric breakdown voltage of 19.1 V, corresponding to a critical field intensity of 3.76 MV·cm^−1^, which is notably lower than the theoretical breakdown field of bulk ZrO_2_. By contrast, the SiO_2_/ZrO_2_ stacked heterostructure exhibited significantly improved dielectric strength, with a breakdown voltage of 24.9 V and a corresponding field strength of 5.78 MV·cm^−1^. The improvement in breakdown performance stems from two synergistic mechanisms: on one hand, the wide bandgap of SiO_2_ (9 eV, higher than ZrO_2_’s 5.8 eV) enables the formation of a more favorable staggered band alignment at the ZrO_2_/SiC interface; on the other hand, the effective reduction in oxygen vacancy density substantially suppresses trap-assisted conduction pathways mediated by defect states, thereby collectively enhancing the dielectric properties. Therefore, the addition of the SiO_2_ layer not only reduced the *I*_ON_EBR_ but also enhanced the *E*_BR_, highlighting the critical role of the stacked structure in optimizing dielectric performance.

A methodical analysis of charge transport phenomena under low-leakage conditions was performed via rigorous numerical simulations across diverse gate electric field regimes. Linear regions of experimental current–voltage profiles were systematically examined to quantify key transport parameters. These parameters were subsequently implemented within computational models aligned with established theoretical frameworks, enabling precise reconstruction of individual conduction pathways.

Current leakage phenomena in MOS capacitor architectures originate from five principal mechanisms: (i) direct quantum tunneling across ultrathin dielectrics, (ii) trap-assisted tunneling (TAT) [35], (iii) Schottky emission [36], (iv) field-enhanced Poole–Frenkel (P-F) conduction [37], and (v) Fowler–Nordheim (F-N) tunneling [38]. Given that direct tunneling occurs primarily in thin oxide films, this study focused on a detailed characterization of TAT, Schottky emission, P-F emission, and F-N tunneling processes. The governing equations for each transport mechanism, along with their associated physical constants and fitting parameters, are summarized in Table 1.

**Figure 8:**
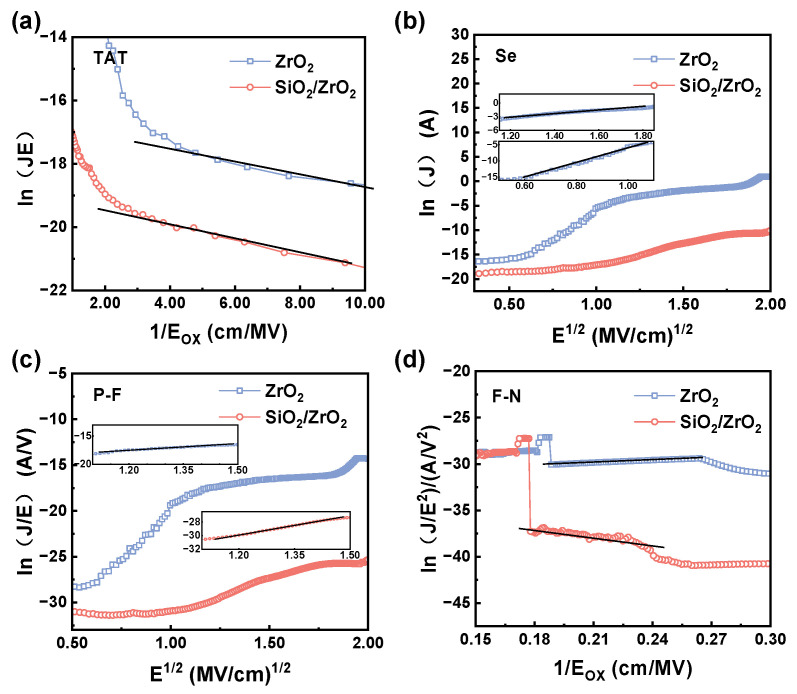
Electric fields of ZrO_2_ and SiO_2_/ZrO_2_ dielectric MOS capacitors for (**a**) TAT, (**b**) Schottky emission, (**c**) F-N emission, and (**d**) F-N tunneling model fitting plots.

The TAT effect occurs when electrons tunnel into trap states within the dielectric film, followed by a transition into the conduction band of the dielectric. As shown in Figure 8a, both ZrO_2_ and SiO_2_/ZrO_2_ dielectric exhibited TAT behavior, indicating the formation of traps within the samples. These traps facilitated the capture and release of electrons at low voltages, creating well-defined TAT conduction pathways [39]. At low-to-moderate bias conditions, TAT is the dominant conduction mechanism, as the applied electric field is insufficient to trigger significant thermal excitation of carriers.

However, as the applied voltage increases, TAT transitions into P-F emission, where charge carriers are thermally excited from bulk dielectric trap states to the conduction band. Under applied electric fields, charge carriers are thermally activated from bulk dielectric trap states to the conduction band, leading to P-F emission [40]. As depicted in Figure 8c, this emission mechanism was primarily observed in both samples under intermediate to strong field conditions, with its dominance increasing proportionally to the magnitude of the applied bias.

Schottky emission refers to the thermally activated transfer of electrons from the semiconductor to the conduction band of the insulator, similar to thermionic emission observed in metal–semiconductor junctions [41]. As shown in Figure 8b, the lower barrier height of ZrO_2_ facilitated Schottky emission, while the higher barrier provided by the SiO_2_ interlayer effectively suppressed this mechanism, reducing leakage current in the SiO_2_/ZrO_2_ stack. Additionally, tunneling effects at moderate electric field strengths caused a sharp increase in current, which contributed to the reduced the dielectric breakdown field intensity (*E*_BR_) and enhanced leakage current [42]. This observation is consistent with previous findings in high-k dielectric stacks, where SiO_2_ interlayers have been demonstrated to effectively block electron injection and suppress Schottky emission [43].

Under elevated gate bias conditions, electrons traversed the triangular energy barrier via quantum tunneling mechanisms, initiating F-N tunneling phenomena under intense electric fields. This behavior was consistently observed in both dielectric configurations, with the characteristic tunneling profiles comprehensively illustrated in Figure 8d. This transition from P-F to F-N conduction is dictated by the electric field strength and the barrier shape at the dielectric interface.

In summary, the leakage current behavior in ZrO_2_ thin films was predominantly governed by multiple mechanisms: TAT, Schottky emission, P-F emission, and F-N tunneling. The SiO_2_/ZrO_2_ stacked structure demonstrated leakage characteristics primarily dominated by TAT, P-F emission, and F-N tunneling, with SiO_2_ interlayer serving as a key modulator of charge transport pathways. Notably, Schottky emission was identified as a major contributor to leakage current variability in ZrO_2_ films, highlighting its distinct role compared to the bilayer configuration. This difference underscores the efficacy of the SiO_2_ interlayer in suppressing specific conduction mechanisms while maintaining overall dielectric integrity.

**Figure 9:**
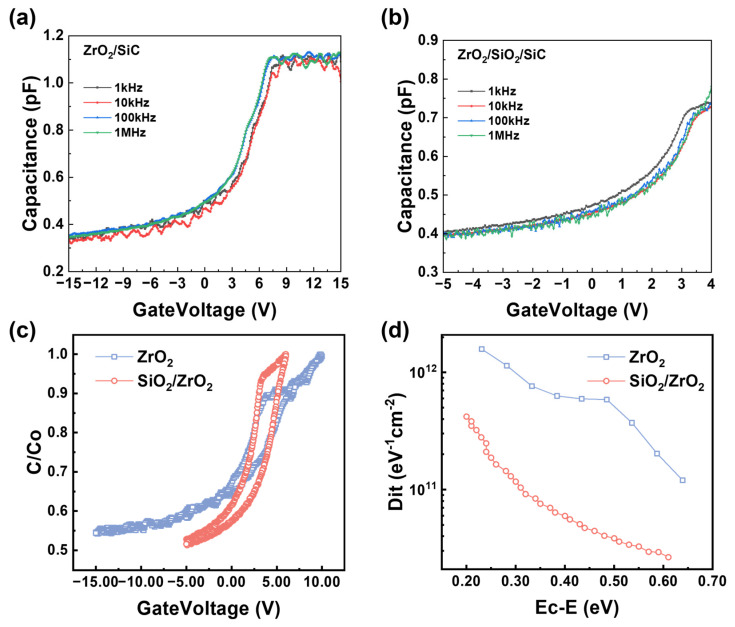
C-V curves of (**a**) ZrO_2_ MOS and (**b**) SiO_2_/ZrO_2_ MOS at 1 kHz, 10 kHz, 100 kHz, and 1 MHz, (**c**) normalized C-V hysteresis curves of MOS capacitors with ZrO_2_ and SiO_2_/ZrO_2_ dielectrics, and (**d**) calculated *D*_it_ plots.

The interfacial properties of MOS capacitors were investigated by measuring the C-V curves of ZrO_2_ and SiO_2_/ZrO_2_ trench MOS capacitors at frequencies of 1 kHz, 10 kHz, 100 kHz, and 1 MHz. As shown in Figure 9a,b, minimal curve scatterings were observed in both samples, indicating the high quality of both gate dielectrics.

To further characterize the interfacial properties, normalized C-V hysteresis measurements were performed at a frequency of 1 MHz. As shown in Figure 9c, under an AC signal of 30 mV, the voltage was swept from negative bias to positive bias and back to negative bias. This cyclic testing protocol effectively captured the dynamic charge trapping and de-trapping behaviors at the dielectric–semiconductor interfaces, with the width of the closed hysteresis loop serving as a key indicator of interfacial quality and its potential degradation or improvement. For the SiO_2_/ZrO_2_ stacked structure, the weighted average method was employed to calculate its effective permittivity ($\varepsilon_{ox}=\frac{d_{{SiO}_{2}}}{d_{total}}\cdot \varepsilon_{{SiO}_{2}}+\frac{d_{{ZrO}_{2}}}{d_{total}}\cdot \varepsilon_{{ZrO}_{2}}$). The effective permittivity of the SiO_2_/ZrO_2_ stack was calculated to be approximately 16 [42]. The flat-band voltage (*V*_FB_ ) values for ZrO_2_ and SiO_2_/ZrO_2_ MOS capacitors were determined as 4.3 V and 3.4 V, respectively [22]. The reduction in flat-band voltage shift highlighted the significant impact of the introduced SiO_2_ layer into the stack.

During high-frequency C-V testing, boundary traps exhibit a delayed response to small AC signal variations due to their slower charge capture and release dynamics. However, these traps can gradually charge or discharge as the DC gate voltage is swept. As a result, when the gate voltage is swept from negative to positive or vice versa, the charge state of these interface traps affects the C-V curve, leading to hysteresis between the high-frequency C-V measurements. This hysteresis occurs because the interface traps are unable to fully charge or discharge at high frequencies, leading to a mismatch between charge accumulation/release and the voltage sweep, thus causing hysteresis [44]. Since the unstacked ZrO_2_ dielectric displayed more significant hysteresis than the SiO_2_/ZrO_2_ stacked dielectric, the lower hysteresis in the stacked structure suggests a reduction in slow trap density, which aligns with the quantitatively lower the low density of interface states (*D*_it_) values obtained through the high–low frequency method.

As shown in Figure 9d, the *D*_it_ values calculated at E_C_-0.2 eV by the high–low frequency method were estimated to be 1.58 × 10^12^ eV^−1^·cm^−2^ for ZrO_2_ and 4.19 × 10^11^ eV^−1^·cm^−2^ for the SiO_2_/ZrO_2_ stack [45]. Thus, both gate dielectric samples contained certain defects, aligning with the TAT mechanism observed under moderate electric fields. The lower *D*_it_ value of the SiO_2_/ZrO_2_ can be attributed to the passivation effect of the SiO_2_ interlayer [46]. Material characterization via XPS and SE previously demonstrated that SiO_2_ forms a stable, uniform interface layer with reduced oxygen vacancy density, which correlates directly with the measured improvement in *D*_it_. On the other hand, the SiO_2_ interlayer serves as a buffer, alleviating the lattice mismatch between ZrO_2_ and the SiC substrate. In the stacked structure, the buffering effect of the SiO_2_ layer isolated ZrO_2_ from direct contact with SiC, mitigating the impact of oxygen vacancies on *D*_it_.

Furthermore, the degradation of channel mobility is primarily influenced by acoustic phonon scattering, surface roughness scattering, and interface defect scattering, with interface defect scattering being the dominant factor. This suggests that the suppression of interface defects via the SiO_2_ interlayer can mitigate mobility degradation, thereby improving channel conduction efficiency and device reliability. The increase in *D*_it_ may also be related to defects introduced during the etching process, where interface quality could be improved through annealing. Nevertheless, the *D*_it_ values of both ZrO_2_ and SiO_2_/ZrO_2_ samples were overall relatively low, indicating good electrical performance.

The *N*_eff_ within the oxide layer can be quantitatively derived from the flat-band voltage (*V*_FB_) [23]. The *N*_eff_ of ZrO_2_ and SiO_2_/ZrO_2_ dielectric MOS capacitors were calculated as −3.63 × 10^12^ cm^−2^ and −1.71 × 10^12^ cm^−2^. As summarized by the electrical and interfacial properties of the MOS capacitors in Table 2, the negative *N*_eff_ values contributed to the forward shift of the flat-band voltage *V*_FB_. The fixed charges or interface traps within the dielectric layer, particularly in the presence of defective oxide layers, can capture or release electrons, thereby influencing the *V*_FB_. The negative fixed charges in the oxide layer cause the accumulation of electrons at the semiconductor–oxide interface when the gate voltage is low, resulting in a forward shift of the *V*_FB_ [47]. For n-type semiconductors, this shift causes an increased accumulation of electrons at the interface. The introduction of a SiO_2_ interlayer into the SiO_2_/ZrO_2_ stacked structure reduced *V*_FB_ shifts and lowered the *N*_eff_, thereby improving the interface quality of the devices. This improvement can primarily be attributed to the SiO_2_ interlayer’s ability to mitigate the effects of interface traps and other charge-related defects within the dielectric stack, resulting in enhanced electrical performance and reliability.

## 4. Conclusions

In summary, this study successfully fabricated MOS capacitors featuring ALD-grown ZrO_2_ and SiO_2_/ZrO_2_ gate dielectric layers on 4H-SiC trench structures. The AFM measurements showed roughnesses of both films less than 1 nm, indicating relatively smooth surfaces. The band gap energy of the SiO_2_/ZrO_2_ film is higher than that of the ZrO_2_ film, as evidenced by both the O 1s energy loss spectrum fitting and the (*αhν*)^2^ versus photon energy analysis. The electrostatic evaluations highlighted distinct variations in dielectric behavior. The ZrO_2_ single-layer configuration displayed an *E*_BR_ of 3.76 MV·cm^−1^, accompanied by *I*_ON_EBR_ reaching 1.94 A·cm^−2^. Mechanistic investigations identified TAT, Schottky emission, PF emission, and F-N tunneling as the principal conduction pathways governing leakage in the monolithic ZrO_2_ system. By comparison, the SiO_2_/ZrO_2_ bilayer architecture achieved an enhanced *E*_BR_ of 5.78 MV·cm^−1^ with *I*_ON_EBR_ reduced to 2.09 × 10^−3^ A·cm^−2^, reflecting a 54% improvement in *E*_BR_ in leakage current compared to the ZrO_2_ monolayer. Within the stacked configuration, leakage mechanisms were predominantly mediated by TAT, PF emission, and F-N tunneling, with Schottky emission effectively suppressed through the interfacial SiO_2_ layer. The C-V hysteresis tests indicated a larger hysteresis in ZrO_2_, consistent with calculated *D*_it_. As a result, the higher interface trap density in ZrO_2_ thin films had a more pronounced impact on the electrical performance. The incorporation of a thin SiO2 interlayer effectively addressed the small bandgap offset, enhancing the bandgap from 5.21 eV to 5.88 eV and reducing *I*_ON_EBR_. However, the etching process induces interfacial defects in the trench structures, which could negatively impact device performance. Post-treatment techniques showed promise in mitigating these interface issues, with future investigations potentially focusing on methods such as annealing to further enhance interface quality. Additionally, research into other high-k materials, such as Y_2_O_3_, Ta_2_O_5_, and La_2_O_3_, could be considered. This comparative study provides a comprehensive evaluation of the electrical behavior of ZrO_2_ and SiO_2_/ZrO_2_ stacked dielectric configurations in SiC trench capacitors, offering valuable insights for optimizing future SiC trench-gate MOSFET designs.

## Figures and Tables

**Table 1:** Conduction plots and analytical model equations of different gate leakage current mechanisms.

Conduction Mechanisms	Conduction Plot	Analytical Model Equations	
TAT	ln(JE) $\;\mathrm{vs}.\;\frac{1}{E}$	$J_{TAT}=\frac{2qc_{T}N_{T}\phi_{T}exp\left(-\frac{D{\phi_{T}}^{\frac{3}{2}}}{E_{ox}}\right)}{3E_{ox}}$	[35]
Schottky emission	ln(J) $\;\mathrm{vs}.\sqrt{E}$	$J_{SE}=A^{*}T^{2}exp\left(\frac{-q\left(\phi_{B}-\sqrt{\frac{qE_{ox}}{4\prod \varepsilon_{ox}}}\right)}{kT}\right)$	[36]
P-F emission	$\ln(\frac{J}{E})$ $\;\mathrm{vs}.\sqrt{E}$	$J_{PF}=qN_{c}\mu E_{ox}exp\left(\frac{-q\left(\phi_{t}-\sqrt{\frac{qE_{ox}}{\pi \varepsilon_{0}k_{r}}}\right)}{kT}\right)$	[37]
F-N tunneling	$\ln(\frac{J}{E^{2}}$ $)\;\mathrm{vs}.\frac{1}{E}$	$J_{FN}=A{E_{ox}}^{2}exp\left(-\frac{B}{E_{ox}}\right)$	[38]

**Table 2:** Electrostatic and interfacial performance metrics of MOS capacitor structures by ZrO_2_ and SiO_2_/ZrO_2_ dielectric.

	*E*_BR_ (MV·cm^−1^)	*V*_FB_ (V)	*E*_BR_ (MV·cm^−1^)	*I*_ON_EBR_ (A·cm^−2^)	*D*_it_ (eV^−1^·cm^−2^) E_C_-0.2 eV	*N*_eff_ (cm^−2^)
ZrO_2_	3.76	4.3	3.76	1.94	1.58 × 10^12^	−3.63 × 10^12^
SiO_2_/ZrO_2_	5.78	3.4	5.78	2.09 × 10^−3^	4.19 × 10^11^	−1.71 × 10^12^

## Data Availability

The original contributions presented in this study are included in the article. Further inquiries can be directed to the corresponding author.
